# Chemotherapeutic Potential of *Carthamus Oxycantha* Root Extract as Antidiarrheal and In Vitro Antibacterial Activities

**DOI:** 10.3390/antibiotics9050226

**Published:** 2020-05-01

**Authors:** Muhammad Ikram, Amany Magdy Beshbishy, Muhammad Kifayatullah, Adedayo Olukanni, Muhammad Zahoor, Muhammad Naeem, Muhammad Amin, Masood Shah, Ahmed S. Abdelaziz, Riaz Ullah, Ramzi A. Mothana, Nasir A. Siddiqui, Gaber El-Saber Batiha

**Affiliations:** 1 Department of Chemistry, Abdul Wali Khan University Mardan, Mardan 23200, Pakistan; ikrambiochem2014@gmail.com (M.I.); naeem@awkum.edu.pk (M.N.); 2 National Research Center for Protozoan Diseases, Obihiro University of Agriculture and Veterinary Medicine, Nishi 2-13, Inada-cho, Obihiro, Hokkaido 080-8555, Japan; amanimagdi2008@gmail.com; 3 Department of Pharmacy, Faculty of Life Sciences, Sarhad University of Science and Information Technology, Peshawar KPK 25000, Pakistan; Kifayatpharma86@yahoo.com; 4 Department of Biochemistry, Redeemer’s University, Ede 00176, Osun State, Nigeria; olukannia@run.edu.ng; 5 Department of Biochemistry, University of Malakand, Chakdara 18800, Pakistan; mohammadzahoorus@yahoo.com; 6 Department of Zoology, University of Karachi, Karachi 75270, Pakistan; aminmuhammad013@yahoo.com; 7 Department of Chemistry, University of Malakand, Chakdara 18800, Pakistan; Masaood.shah20@gmail.com; 8 Pharmacology department, Faculty of Veterinary Medicine, Zagazig University, Zagazig 44519, Egypt; asabdelaziz@vet.zu.edu.eg or; 9 Department of Pharmacognosy, College of Pharmacy, King Saud University, P.O. Box 2457, Riyadh 11451, Saudi Arabia; rullah@ksu.edu.sa (R.U.); rmothana@ksu.edu.sa (R.A.M.); nsiddiqui@ksu.edu.sa (N.A.S.); 10 Department of Pharmacology and Therapeutics, Faculty of Veterinary Medicine, Damanhour University, Damanhour 22511, AlBeheira, Egypt

**Keywords:** antibacterial activity, antidiarrheal, methanolic extract, *Carthamus oxycantha*, MIC (minimum inhibitory concentration)

## Abstract

Our research work was designed to investigate the curative and preventive effects of *Carthamus oxycantha* root extract against diarrhea and microorganisms. For the antibacterial experiment, the agar well diffusion method was used against standard bacteria *Staphylococcus aureus*, *Escherichia coli*, *Pseudomonas aeroginosa,* and *Salmonella typhi,* while for the assessment of antidiarrheal activity, castor oil and the magnesium sulfate-induced diarrhea method was used on albino, laboratory-bred (BALB/c) mice at a dose rate of 200 and 400 mg/kg (body weight, b.w) orally. The methanol extract of *C. oxycantha* significantly (*p* < 0.001) decreased the frequency of defecation, and wet stools in a dose depended on the manner of after receiving magnesium sulfate (2 g/kg (b.w)) and castor oil (1.0 mL/mice). Furthermore, the extract of *C. oxycantha* showed concentration-dependent antimicrobial properties against *S. aureus* followed by *S. typhi*, *E. coli,* and *P. aeroginosa* bacterial strains, with inhibitions ranging from 10.5–15 mm. These findings show significant results that *C. oxycantha* is effective as an antidiarrheal and antibacterial agent. However, further works are needed to establish its mode of action.

## 1. Introduction

Plants are traditionally used for the prevention of various diseases [1]. It has been widely recorded that about 80 percent of the world’s population depends on botanicals for the treatment of several diseases [2,3]. About 122 compounds currently used in medicine are derived from “ethnomedical” plant sources. The utilization of plants in alternative medicine is important evidence of plants’ role in the world economy [4,5]. Asteraceae is one of the largest families of flowering plants. It includes more than 1000 genera and about 10,000 species distributed over all parts of the world [6]. The genus *Carthamus*, one of the family Asteraceae, includes 25 species. Reviewed literature showed that many of *Carthamus* species have been used to treat many diseases and complaints such as cough, typhoid fever, throat disorders, cardiovascular problems, swelling, menstrual disorders, anti-inflammatory, antioxidant, calcium antagonist, and anticoagulant and also used in folk medicine as a sedative and anticancer herbal product [7]. 

*Carthamus oxyacantha,* like other species in its genera, has shown diverse biological activities ranging from local to systemic actions [8]. The seeds and oils of the plants are known to be of medicinal value, while its young leaves are used as a vegetable. The *C. oxycantha* seeds are a good source of oil for cooking, and its fruit is used as bird feed. In essence, *C. oxycantha* herbage and the straw are valuable as fodder in many countries. The young leaves of *C. oxyacantha* are utilized as a vegetable, while seeds are used in cooking, serving as an alternative for true saffron, or a natural food colorant. Two types of oils are commonly found in this species: oleic oil and linoleic oil. Traditionally, the flowers of *C. oxycantha* are used in the treatment of rheumatism, cerebral thrombosis, male infertility, bacteria, diarrhea, and bronchitis, along with jaundice and constipation [9,10]. Medicinally, the herbage *C. oxyacantha* is used for wound-healing and anti-inflammatory purposes. It also has the potential for antimicrobial, antioxidant, and antiworm actions. Two major pigments are found in its flowers: carthamidin and the carthamin. Its seeds and flowers have been reported with compounds like glycosides, serotonin, flavonoids, and sterols [11,12].

Diarrhea is one of the major healthcare burdens and one of the well-known causes of mortality and morbidity amongst the children globally [13]. It is described as the loss of stomach control by animal/human, characterized by bowel movements and loose and watery stools. Infectious diarrhea occurs when certain bacteria enter the body through contaminated food or water and cause diarrhea. The main causative microbial factors responsible for the development of diarrhea in humans are *Escherichia coli, Staphylococcus aureus, Shigella flexneri,* and *Salmonella typhi*, followed by *Candida albicans* [14]. In a recent study involving 259 diarrheal patients of ages five years and below, Chuang et al. [15] observed 15% *P. aeruginosa* enterocolitis and 19% *P. aeruginosa*-related diarrhea. Despite the widespread knowledge of bacteria as causes of diarrhea, diarrhea is still being induced in animal studies using chemical methods. 

Several methods are used to induce diarrhea in animals, such as magnesium sulfate and castor oil. Castor oil-induced diarrhea is due to the contraction of smooth muscles that decrease the fluid absorption and increase the secretion in the colon followed by the small intestine [16]. The active constituent in the induction is ricinoleic acid, which acts as an irritant purgative that stimulates the sensory nerve-endings in the small intestine, the Auer Bach reflex [17], along with intestinal Na^+^, K^+^-ATPase inhibition, thus reducing the absorption of routine fluid in the body [18]. On the contrary, the mode of action of magnesium sulfate is with the aid of ulcerated cholecystokinin release. These methods provide techniques for inducing diarrhea without little ethical issues, thus making clinical studies with herbal medicines possible.

Most pediatric diarrheas occur in a rural area, where its treatment depends on herbal medicines. In the last decades, the use of herbal medicines is increasing rapidly, because they have no side-effects, they are easily accessible, and of low cost. Keeping in view the medicinal importance of plants, the present work was proposed to evaluate the antidiarrheal and in vitro antibacterial activities of *C. oxycantha* in experimentally induced diarrhea and standard bacterial strains.

## 2. Materials and Methods

### 2.1. Collection, Identification, and Extraction of Plant Material

The whole plant of *C. oxycantha* was collected in October 2015 from the local area of District Dir, KPK, Pakistan and was identified by the Department of Botany, University of Malakand. The plant was washed with running water, and the roots were separated and dried in a shady place for 4 weeks. The roots after drying were grounded into a coarse powder with the grinding machine [19,20]. The ground materials (powder) were kept in airtight plastic bottles until further use. After that, 500 g of the ground roots were subjected to maceration for the process of extraction using methanol as a solvent. The powders were soaked for 14 days with constant stirring, filtered with the muslin cloth, and then, finally, with filter paper. The resultant extract was then concentrated under reduced pressure via a rotary evaporator and placed in a clean hood for evaporation until dryness [21,22]. The dried crude extract was kept in the refrigerator at a temperature of 5 °C for further use. 

### 2.2. Drugs, Chemicals, and Instruments

The drugs, chemicals, and instruments that were used in the present study were loperamide (Sigma Aldrich, Steinheim, Germany), magnesium sulfate (Sigma Aldrich), castor oil, methanol (BDH Chemicals, London, England), SS agar (Liofilchem, Roseto degli Abruzzi, Italy), and metabolic cages.

### 2.3. Test Microorganisms

Standard bacterial strains e.g., *E. coli* (ATCC® 25922™), *S. typhi* (ATCC®19430™), *S. aureus* (ATCC^®^25923^™^)*,* and *P. aeruginosa* (ATCC® 15442™) were used for the antibacterial studies [23].

### 2.4. Test Animals

Albino, laboratory-bred (BALB/c mice of females and males having a normal weight range from 25–30 g [24] were employed in this study that was procured from the National Institute of Health, Islamabad. The animals were kept in the plastic cages at the animal house of the University of Malakand, Pakistan under standard environmental conditions (23–25 °C) [25,26]. The night before the experiment, the animals fasted overnight, and further treatments were according to the rules mentioned in the 2008 bylaws of The University of Malakand (Scientific Procedures Issue I). The protocols involving the animals used were sanctioned by official bodies (Ethical Committee) of the University of Malakand, KPK, Pakistan.

### 2.5. Acute Toxicity Study

Organisation for Economic Co-operation and Development (OECD) guideline 423 was used for determining the acute toxicity study with some modifications [27]; for instance, the maximum single test dose of 2000 mg/kg (body weight, b.w) was employed for female adult BALB/c mice. The animals were distributed into two groups consisting of six animals each. Group I was the control; group II received a single dose of methanol extract of *C. oxycantha* 2000 mg/kg (b.w) orally and was kept as a tested group. Before administering the test drug, each mouse was weighed, and the dose for it was determined based on its weight. The treated animals were monitored for any mortality and toxic effects within the first 4 h, after 3 days, and then twice daily for 14 days. Behavioral changes and other parameters such as body weight, urination, water intake, respiration, food intake, tremors, convulsions, temperature, constipation, and changes to skin and eye colors were also monitored.

### 2.6. Dose Selection

Two doses of methanol extract of *C. oxycantha* were selected according to 1/10th of the maximum toxic dose and were diluted in normal saline. 

### 2.7. Experimental Design

#### 2.7.1. Castor Oil-Induced Diarrhea

The antidiarrheal activity was performed on BALB/c mice in castor oil-induced diarrhea with some modifications in the protocols [28]. Animals were randomly assigned into four groups of five animals each. Group I was the control and received normal saline, while group II was the standard that received (loperamide) at a dose rate of 50 mg/kg (b.w). Groups III and IV were the tested groups that received the methanol extract at a dose rate of 200 mg/kg and 400 mg/kg (b.w), respectively. After the treatment of respective drugs, each animal was put in separate cages laid with papers for collecting the fecal mass. Diarrhea was induced by oral administration of castor oil (1 mL/mice). The methanol extract and loperamide were given 1h before the oral administration of the castor oil; the time is taken for the first feces excretion, and the total number of fecal outputs within 6 h of administration was recorded. The inhibition of defecation was calculated using the following equation:

% inhibition = (Mo–M)/Mo × 100,

where Mo = mean defecation of control and M = mean defecation of the experimental group.

Loperamide, the standard drug used, slows intestinal motility and alters the bowel water and electrolyte movement. It is a potent opiate receptor agonist in the gut wall where it inhibits the release of acetylcholine and prostaglandins, thereby reducing propulsive peristalsis and increasing the intestinal transit time. The drug has also been reported to increase the activity of the anal sphincter [29]. It thus antagonizes the diarrheal activity induced with castor oil [30].

#### 2.7.2. Magnesium Sulfate-Induced Diarrhea

The effect of *C. oxycantha* on magnesium sulfate-induced diarrhea was also determined on adult healthy BALB/c mice [31]. After overnight fasting, the animals were distributed into 4 groups of 5 animals each. Group I, the control, received only normal saline. Group II, the standard, received loperamide at a dose rate of 20 mg/kg (b.w), while groups III and IV, which were the test groups, received a methanolic extract of *C. oxycantha* at a dose rate of 200 and 400 mg/kg (b.w). Sixty minutes after treatment of the respective drug, all the animal groups were treated orally with magnesium sulfate at a dose rate of 2 g/kg (b.w). The frequency of defecation and the fecal material was again noted for up to 4 h. The mice were in transparent (clear) cages with pre-weighed plastic dishes for feces collection at the bottom of all cages. The weights of the plastic dishes were recorded and compared to that of the control before and after defecation.

#### 2.7.3. Test Microorganisms and Growth Conditions

Four standard bacterial strains: *S. aureus*, *S. typhi, E. coli,* and *P. aeruginosa* were used in this study. Agar nutrient medium was used for the growth of bacterial strains and was allowed to stand for a period of 24 h at 37 °C. Nutrient agar was added to a conical flask that already contained distilled water. The nutrient agar powder and distilled water were mixed in (proper ratio). The aqueous solution was made by incorporating an amount of 20 gm of agar nutrient in 1000 mL of distilled water with constant shaking for 6 min. The solution was then sterilized and transferred to Petri dishes for the inoculation of bacterial strains.

#### 2.7.4. Well Diffusion Method

The antibacterial activity of *C. oxycantha* was determined individually by the agar well diffusion method [32]. Twenty milliliters of molten nutrient agar was poured into each of the Petri dishes and allowed to solidify. Overnight, a bacterial broth, standardized to 0.5 McFarland, was spread on the dry nutrient agar and spread using a spreader pre-sterilized in ethanol and flamed. With the aid of a sterile cork-borer, five 6-mm holes, about 5 cm apart, were made in the nutrient agar. Three of the wells were filled with 200 µL of the *C. oxycantha* plant extract dissolved in sterile distilled water, one well with the water only (the negative control), and the last with 1% standard antibiotic, doxycycline (the positive control) was dispensed into the wells in triplicates. The antibacterial activities were determined after incubation for 24 h period at 37 °C as the diameter of the inhibition zone. The zones of inhibition observed with the extract were compared with that of the standard antibiotic, doxycycline. The experiment was done in triplicates. The measured doxycycline inhibition zones’ diameters were subsequently matched with the respective standard zones’ diameters [33] for *S. aureus*, *S. typhi, E. coli,* and *P. aeruginosa* [34]. The *C. oxycantha* zone of inhibition from 9-14 mm in diameter was taken as a positive antibacterial activity based on those previously reported for *Carthamus caeruleus* L [35] and *Carthamus tinctorius* [36]. The fold change in the doxycycline and *C. oxycantha* zone of inhibition was measured by the following equation: (B−A)/A, where the *C. oxycantha* and doxycycline zone of inhibition is A and B, respectively. 

#### 2.7.5. Determination of Minimum Inhibitory Concentration (MIC) 

Solutions of *C. Oxycantha* extract at varied concentrations of 1.0 mg/mL, 2.0 mg/mL, 3.0 mg/mL, 4.0 mg/mL, and 5.0 mg/mL were introduced to molten agar plates and incubated for 24 h at 37 °C. The plates were then inoculated with the specific bacterial strains, incubated at 37 °C for 24 h, and the minimum inhibitory concentration (MIC) of the extract was determined against the selected bacterial strains. The MIC of the extract against the bacterial strains was found to be 0.5 mg/mL, which shows that, while increasing the concentration, their activity also increased [37].

### 2.8. Data Analysis

All experiments were conducted in triplicates or more replicates, and data were presented as mean ± SEM; values were considered significantly different at 95% confidence, i.e., *p* < 0.05. In addition, a one-way ANOVA test followed by Turkey’s multiple comparisons was employed for the antidiarrheal studies [38].

## 3. Results

### 3.1. Acute Toxicity Study

No mortality and any treatment-related toxic effects were observed after a single administration of *C. oxycantha* extract at 2000 mg/kg (b.w) limited dose to the female mice. The general observation of the tested groups did not show any visual changes in breathing, food intake, water consumption, behavior, skin, and temperature during the fourteen days of the study. Hence, the tested drug was considered nontoxic even at a high dose level of 2000 mg/kg (b.w). However, laziness, itching, and sedation were found for the first 12 h, which became normal on the sixth day after receiving *C. oxycantha* when compared to the control group. The parameters that were analyzed during the 14 days of the study are shown in Table 1. 

### 3.2. Castor Oil-Induced Diarrhea

The antidiarrheal activity of the methanolic extract of *C. oxycantha* and standard drug in castor oil-induced diarrhea are given in Table 2. A significant decrease (*p* < 0.001) in the mean number of defecations was noted in the methanolic extract and loperamide-receiving groups while compared to control. The attenuation in the mean number of defecations with *C. oxycantha* at a dose rate of 200 mg/kg (b.w) was (*p* < 0.05) 7.33 ± 1.76. Similarly, the attenuation in the mean number of defecations in the animal group that received a *C. oxycantha* extract at the dose rate of 400 mg/kg (b.w) was 5.20 ± 1.15, which seemed to be more significant (*p* < 0.01) as a match to the control group. Furthermore, the mean number of defecations with loperamide at the dose of 50 mg/kg (b.w) was more significant (*p* < 0.001) at 3.66 ± 0.66. The results indicate that the percentage of attenuation in the mean number of defecations by the extracts of both doses was 21.43% and 44.26%, respectively. Additionally, the percentage of attenuation in the mean number of defecations with loperamide was 60.77%. The latent period for *C. oxycantha* increased significantly (*p* < 0.001) as compared to the control group.

### 3.3. Magnesium Sulfate-Induced Diarrhea

The antidiarrheal activity of the *C. oxycantha* extract was also evaluated in the magnesium sulfate-induced diarrhea method. The results presented in Table 3 show that the groups that received *C. oxycantha* at the dose rates of 200 and 400 mg/kg (b.w) produced significant and dose-dependent (*p* < 0.01) attenuations in the total number of stools and wet stools as compared to the control. The mean number of defecations with the *C. oxycantha* extract at a dose rate of 200 mg/kg (b.w) was 7.66 ± 0.33, while the mean number of defecations with 400 mg/kg (b.w) was 6.00 ± 0.57 compared to the control group. The mean number of defecations produced by the standard drug was 4.20 ± 0.33. The percentage of inhibition of defecations with the methanolic extract at doses 200 and 400 mg/kg (b.w) were 30.36% and 45.45%, respectively, while that of the loperamide standard, at dose 20 mg/kg (b.w), was 61.81%.

### 3.4. Well Diffusion Method 

**Figure 1:**
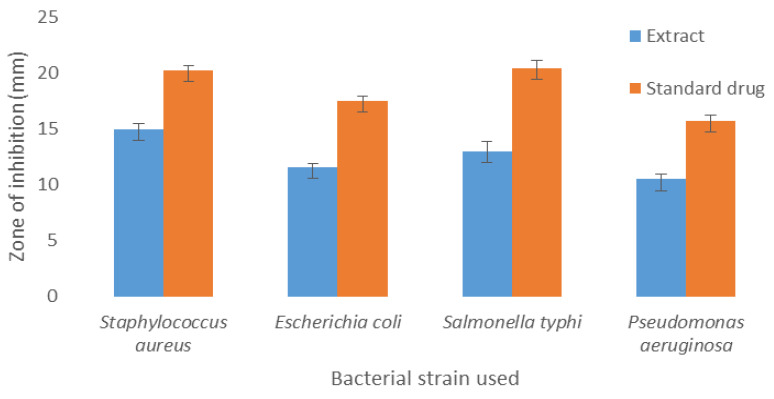
Graphical representation of the antibacterial activity of the methanolic root extract of *Carthamus oxycantha*.

The antimicrobial activity of *C. Oxycantha* root extract was determined as a zone of inhibition against *S. aureus, E. coli, S. typhi,* and *P. aeroginosa* growth. This was compared with doxycycline as a positive standard in which *S. aureus, E. coli*, *S. typhi,* and *P. aeroginosa* were susceptible to doxycycline with zones of inhibition (mm) that were 20.25, 17.5, 20.5, and 15.75, respectively (Figure 1). The result revealed that the methanolic extract has a significant antibacterial potential against all bacterial strains, and the zones of inhibition (mm) were 15, 11.6, 13, and 10.5 against *S. aureus E. coli*, *S. typhi,* and *P. aeroginosa*, respectively (Table 4). Moreover, 0.35–0.57-fold changes in the zones of inhibition (mm) for *C. Oxycantha* were compared with doxycycline, which was used as a reference drug; additionally, the *C. Oxycantha* zone of inhibition that in all bacterial species ranged from 10.5–15 mm is considered as susceptible.

### 3.5. Determination of Minimum Inhibitory Concentration (MIC) 

The MIC values often determined to investigate the efficacy of a given antimicrobial agent against some known pathogens. The MIC of the methanol extract against the bacterial strain was found to be 5 mg/mL. The study also showed that the antibacterial activities of the extract increased with an increase in concentration, which shows that, when the concentration of extract increases, their activity also increases.

## 4. Discussion

The present research was proposed to find out the antidiarrheal effects of the *C. oxycantha* root extract against castor oil and magnesium sulfate-induced diarrhea, as well as its antibacterial activity. The result of this study shows that *C. oxycantha* extract produced dose-dependent antidiarrheal effects in the castor oil-induced diarrhea method. 

The antidiarrheal effect of *C. oxycantha* was intimately analogous to loperamide, which is presently, widely and effectively used as an antidiarrheal drug globally. The *C. oxycantha* extract adequately counteracted the increase in electrolyte secretion, perhaps due to the anti-electrolyte permeability action. Rode et al. [39] and Yacob et al. [40] attributed the antidiarrheal activities of *Diospyros malabarica* bark extract and *Ajuga remota* to anti-electrolyte permeability action. Additionally, the extracts also caused a significant inhibition in the accumulation of intestinal fluids and intestinal contents [39]. We hypothesized that the methanolic extract of *C. oxycantha* inhibits the secretion of water into the intestinal lumen, an effect that is partially mediated by muscarinic receptors and α2-adrenoceptor systems [40]. The antidiarrheal properties of plant-based drugs are due to the presence of saponins, triterpenoids, flavonoids, tannins, alkaloids, and reducing sugars [41]. The phytochemical analysis on *C. oxycantha* revealed the presence of alkaloids, flavonoids [40,42], triterpenoids, carbohydrates, tannins, and phenols that may mediate the antidiarrheal property [43,44,45]. For determining the antibacterial activity of *C. oxycantha* against bacterial strains *P. aeroginosa, E. coli*, *S. aureus,* and *S. typhi*, the well diffusion method was adopted. Minimum inhibitory concentration was also determined for the selected bacteria. The lowest concentration having no turbidity was determined and was recorded as the MIC value. *C. oxycantha* showed the greater antibacterial activity against *S. aureus,* followed by *S. typhi*, *E. coli,* and *P. aeroginosa.* The minimum inhibitory concentration of extracts for the selected bacterium strains was 5 mg/mL. However, the extracts overall showed better antibacterial activities as compared to doxycycline, which is the standard antibiotic; that may be due to the purity of the chemical constituents. According to Raza et al. [46], the ethanolic crude extract of the *C. oxyacantha* plant showed a broad spectrum of antimicrobial activity against all the selected bacterial strains.

## 5. Conclusions

In this present study, extracts of the *C. oxycantha* root show potential against diarrhea in castor oil and magnesium-induced diarrhea and exhibited antibacterial activities (ZOI of between 10.5–15 mm) against the standard strains. As an alternative source, efforts should be made towards deploying *C. oxycantha* root extract as an antidiarrheal and antimicrobial agent [47]. However, further research is needed to unravel the mechanisms of action of these effects and to identify the bioactivity of the various phytochemicals responsible for the medicinal values of *C. oxycantha* [48,49,50].

## Figures and Tables

**Table 1:** Effect of *Carthamus oxycantha* extracts for 14 days on the behavioral and general appearances of the treated and control groups.

Observation	Control	2000 mg/kg (b.w)
Digestion	N/O	N/O
Bodyweight	N	N/C
Itching	N	O
Food intake	N	N
Skin	N/E	N/E
Laziness	N/P	P
Sedation	N/E	O
Diarrhea	N/P	N/P
General physique	N	Lethargy
Coma	N/P	N/P
Eye color	N/E	N/E
Death	Alive	Alive

N/O: not observed, O: observed, N/P: not present, N/E: no effect, N: normal, N.C: no change, and P: present. b.w: body weight.

**Table 2:** Antidiarrheal activity of *C. oxycantha* extracts on castor oil-induced diarrhea.

Group	Dose (mg/kg (B.w))	Mean ± SE		% of Inhibition
		Latency	Defecation	
Normal Control	10 mL/kg	0.50 ± 0.45	9.33 ± 1.45	-
*Carthamus oxycantha*	200	1.75 ± 0.20	7.33 ± 1.76 *	21.43
*Carthamus oxycantha*	400	2.10 ± 0.50	5.20 ± 1.15 **	44.26
Standard (Loperamide)	50	3.47 ± 1.20	3.66 ± 0.66 ***	60.77

Data are represented as mean ± SEM, N = 05, * *p* < 0.05, ** *p* < 0.01, and *** *p* < 0.001. Significant when compared with control. The statistical test employed was a one-way ANOVA test followed by a Turkey’s multiple comparisons.

**Table 3:** Antidiarrheal activity of the methanolic extract in the magnesium sulfate-induced diarrheal test.

Group	Dose (mg/kg (b.w))	Mean ± SEM		% of Inhibition
		Latency	Defecation	
Normal control	10 mL/kg	0.75 ± 0.10	11.0 ± 1.0	-
*Carthamus oxycantha*	200	2.20 ± 0.16	7.66 ± 0.33 *	30.36
*Carthamus oxycantha*	400	2.81 ± 0.19	6.00 ± 0.57 **	45.45
Standard (Loperamide)	50	3.41 ± 0.25	4.20 ± 0.33 ***	61.81

Data are expressed as mean ± SEM, N = 05, * *p* < 0.05, ** *p* < 0.01, and *** *p* < 0.001. Significant as matched to the control. The statistical test employed was a one-way ANOVA test followed by a Turkey’s multiple comparison test.

**Table 4:** Antibacterial activity of the methanolic extract of the *C. Oxycantha* root.

Microorganism	Interpretation of Doxycycline Zone Diameters (mm)			ZOI (mm) for the Standard Antibiotic (Doxycycline)	ZOI (mm) for the Methanolic Extract of *C. oxycantha*	Fold Change between the Doxycycline and *C. oxycantha* Zone of Inhibition	% Age Yield ZOI
	R ≤	I	S ≥				
*Staphylococcus aureus*	12	13–15	16	20.25 ± 0.420(S)	15.0 ± 0.500(S)	0.35	74.07%
*Escherichia coli*	10	11–13	14	17.50 ± 0.500(S)	11.6 ± 0.208(S)	0.5	66.28%
*Salmonella typhi*	10	11–13	14	20.50 ± 0.688(S)	13.0 ± 0.866(S)	0.57	63.41%
*Pseudomonas aeruginosa*	NA	NA	NA	15.75 ± 0.500	10.5 ± 0.500(S)		66.6%

R: resistant, I: intermediately susceptible, S: susceptible, ZOI: zone of inhibition.
